# Genetic structure and diversity of green turtle (*Chelonia mydas*) in the Gulf of Thailand

**DOI:** 10.14202/vetworld.2024.37-49

**Published:** 2024-01-04

**Authors:** Poommate Chomchat, Worata Klinsawat, Kaitkanoke Sirinarumitr, Natnaree Inthong, Theerapol Sirinarumitr

**Affiliations:** 1 Doctor of Philosophy (Program Veterinary Clinical Studies), Faculty of Veterinary Medicine, Kasetsart University, 50 Ngamwongwan Road, Chatuchak, Bangkok, 10900, Thailand; 2 Conservation Ecology Program, School of Bioresources and Technology, King Mongkut’s University of Technology Thonburi, Bang Khun Thian Chai Thale Road, Tha Kham, Bang Khun Thian, Bangkok, 10150, Thailand; 3 Kasetsart University Veterinary Teaching Hospital, Faculty of Veterinary Medicine, Kasetsart University, 50 Ngamwongwan Road, Chatuchak, Bangkok, 10900, Thailand; 4 Department of Veterinary Nursing, Faculty of Veterinary Technology, Kasetsart University, 50 Ngamwongwan Road, Chatuchak, Bangkok, 10900, Thailand; 5 Department of Pathology, Faculty of Veterinary Medicine, Kasetsart University, 50 Ngamwongwan Road, Chatuchak, Bangkok, 10900, Thailand

**Keywords:** genetic diversity, green turtle, Gulf of Thailand, phylogeographic

## Abstract

**Background and Aim:**

The International Union for the Conservation of Nature and Natural Resources lists the green turtle as endangered. Green turtle nesting behavior in the Gulf of Thailand has decreased to <50% of the 1995 level. The population structure of green turtles in the Gulf of Thailand has not yet been studied. This study aimed to characterize the genetic diversity of green turtles in the Gulf of Thailand based on comparisons of mitochondrial DNA (mtDNA) control region with sequences of Indo-Pacific management units (MUs) and rookeries, to investigate population structures, and to explore phylogeographic relationships.

**Materials and Methods:**

Blood samples (1 mL each) from 91 stranded green turtles were collected from four parts of the Gulf of Thailand (eastern, upper, central, and lower). The control mtDNA region was amplified by polymerase chain reaction using LCM15382 and H950 primer. The obtained 384-bp or 770-bp sequences were analyzed for haplotype, clade, and haplotype and nucleotide diversities and were used to construct a phylogenetic tree and haplotype network diagram, respectively. In addition, we analyzed genetic differentiation within and among populations of green turtles in the Gulf of Thailand and between green turtles in the Gulf of Thailand and other Indo-Pacific MUs and rookeries.

**Results:**

In total, 12 (based on 384 bp) or 13 (based on 770 bp) haplotypes and two clades (clades VII and VIII) were identified, with nine or 10 haplotypes belonging to clade VIII and three haplotypes belonging to clade VII. Of the new haplotypes, four or five were identified and classified as clade VII (two haplotypes, for both fragment lengths) and clade VIII (two or three haplotypes, for 384 bp or 770 bp fragments, respectively). The overall haplotype and nucleotide diversity of green turtles in the Gulf of Thailand were high (0.755 ± 0.039 and 0.01146 ± 0.00248, respectively). Based on the analysis of molecular variance, green turtles in the Gulf of Thailand could be divided into two subpopulations (UC-Eastern Gulf of Thailand [UC-EGT] and lower Gulf of Thailand [LGT]). Comparisons with other MUs and rookeries in the Indo-Pacific showed that UC-EGT was not genetically different from the Peninsular Malaysia and Eastern Taiwan (Lanyu) MUs and the Terrangganu and Mersing rookeries, and LGT were not genetically different from Peninsular Malaysia, Sipadan, Brunei Bay, Eastern Taiwan (Lanyu), Scott Reef and Browse Island, and Gulf of Carpentaria MUs and the Perak, Perhentain Island, Redang, Pahang, and Vietnam rookeries.

**Conclusion:**

To the best of our knowledge, this is the first report to identify the haplotypes and clades of green turtles in the Gulf of Thailand and to show that the populations in the Gulf of Thailand not only present high genetic diversity but also have haplotypic endemism. Longer mtDNA fragments (770 bp) increased the resolution of the stock structure. Clade VII is a unique clade not only for Japan but also for Thailand and Malaysia, and CmP82 is a unique haplotype for both the Gulf of Thailand and Malaysia. Conservation and management of these populations are important to preserve the genetic diversity, biological diversity, and evolutionary potential of green turtles in the Gulf of Thailand.

## Introduction

There are five species of sea turtles in Thailand: Green (*Chelonia mydas*), hawksbill (*Eretmochelys imbricate*), olive ridley (*Lepidochelys olivacea*), loggerhead (*Caretta caretta*), and leatherback (*Dermochelys coriacea*). Green, hawksbill and olive ridley turtles are the most commonly found sea turtles. Green turtles are found in both the Gulf of Thailand and the Andaman Sea; however, hawksbill and olive ridley turtles are only found in the Andaman Sea [1]. Green turtle nesting behavior in the Gulf of Thailand has decreased to <50% of the 1995 level [2]. Some green turtle populations, especially in Southeast Asia, have declined due to various anthropogenic threats (by-catch in fisheries, legal and illegal harvest of turtles), collection of eggs, marine pollution, habitat degradation by coastal development, or altered habitat quality at nesting beaches and feed grounds, including an increase in sea level due to climate change [3]. Thus, green turtle is listed as endangered by the International Union for the Conservation of Nature and Natural Resources [4]. Female green turtles are philopatric and have strong nest-site fidelity; however, this is not perfect, resulting in the formation of new colonies [5]. After hatching, the hatchlings move toward the sea and reach the pelagic habitat (pelagic stage) through both active swimming and ocean current. Subsequently, juvenile green turtles were recruited to the neritic habitat (neritic stage) [6]. Juvenile green turtles are omnivores; however, they become herbivores when mature [1]. Globally, there are two phylogeographic clusters of green turtles: Atlantic/Mediterranean and Indo-Pacific [7].

Conservation of green turtles requires knowledge of the rookeries component (composed of regional breeding populations) and the extent to which these populations contribute to the regional foraging ground [8]. However, it is challenging because green turtles can migrate long distances. The population dynamics and population structure of green turtles can be examined using mark and recapture, satellite telemetry, mitochondrial DNA (mtDNA) sequencing, and microsatellite analyses [9]. Mark recording and satellite tracking provide direct evidence of movement, whereas satellite tracking provides information on demographics, sites, and migration routes. Nevertheless, it provides only individual information and is biased toward intensively studied locations [10]. Maternally inherited mtDNA has been used to study the population structure of migratory marine animals and to identify distinguishable stocks for management [11]. The mtDNA control region is a hypervariable, non-coding region of the mtDNA that provides information to detect population differentiation in sea turtles [7]. Studies have used the 384 base pair (bp) fragment of the control region; more recently, studies have used the 770 bp fragment of the entire control region, which provides higher resolution than the 384 bp fragment in certain rookeries [12]. Microsatellites are highly polymorphic nuclear DNA markers that have been used to study identity, paternity, regional geographic structuring, and male-biased gene flow [13]. A major limitation of this technique is the genotyping of dead hatchlings inside the nest to nesting females because all hatchlings from a certain nest share the same mother but not necessarily the same father [14].

In Thailand, two studies using satellite telemetry for green turtles in both the Gulf of Thailand and the Andaman Sea [15, 16] have been reported on the population dynamics and population structure of green turtles; however, these studies were conducted on a very small scale. Globally, green turtles have been classified into 11 clades and hundreds of haplotypes, covering 127 rookeries and 58 management units (MUs) in 12 geographical regions using the mtDNA control region [7]. In Thailand, mtDNA control regions of green turtles in both the Gulf of Thailand and the Andaman Sea [17] have been reported. However, the primers used in the present study were not the same as the primers commonly used in other studies and were peripheral to other studies. Therefore, the mtDNA control region sequences (438 bp) obtained in the previous study cannot be used for analysis or comparison with the sequences, haplotypes, and clades identified in the other studies.

Thus, the aim of the current study was to characterize the genetic diversity of green turtles in the Gulf of Thailand using 384 and 770 bp of mtDNA control region and compare it with Indo-Pacific sequences of MUs and rookeries.

## Materials and Methods

### Ethical approval

This study was approved by the Animal Ethics Committee of the Faculty of Veterinary Medicine, Kasetsart University, Thailand (ACKU63-VET-021) and was in accordance with the Guidelines for Animal Care under the Ethical Review Board of the Office of National Research Council of Thailand for conducting scientific research.

### Study period and location

The study was conducted from March 2020 to December 2022 at the Faculty of Veterinary Medicine, Kasetsart University, Thailand.

### Sample collection

**Figure-1:**
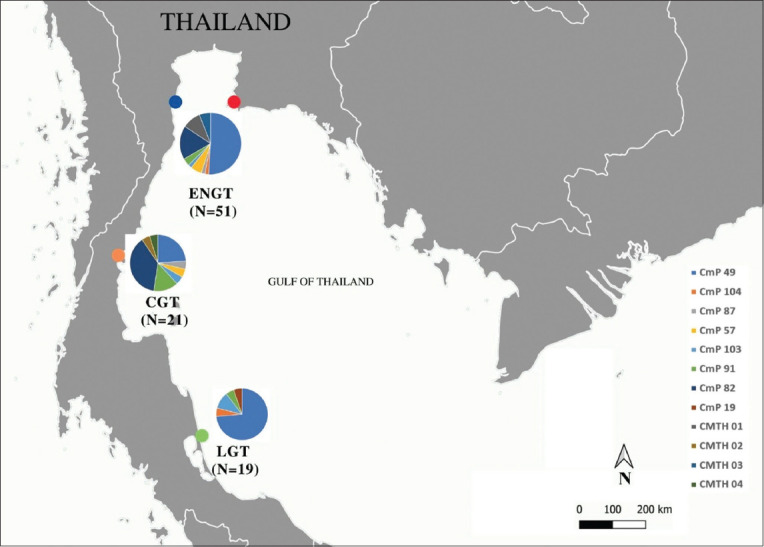
Collection sites (Eastern Gulf of Thailand [red dot], upper Gulf of Thailand [blue dot], center Gulf of Thailand [orange dot] and lower Gulf of Thailand [green dot] in this study. Pie charts indicate relative proportion of each haplotype (based on 384 bp mitochondrial DNA control region) at each collection site with color corresponding to specific haplotypes.

A blood sample (1 mL) was collected from each of 91 stranded green turtles (48 samples from the Marine and Coastal Resources Research Center, the Eastern Gulf of Thailand (EGT) at 12.690N, 101.700E; three samples from the upper Gulf of Thailand (UGT) at 13.500N, 100.260E; 21 samples from the central Gulf of Thailand (CGT) at 10.6990N, 99.240E; and 19 samples from the lower Gulf of Thailand (LGT) at 7.160N, 100.580E), as shown in Figure-1. Each sample was collected from the dorsal cervical sinus, placed in an ethylenediaminetetraacetic acid tube, and kept at -20°C until analysis. After blood collection, the curved carapace length (CCL) and curved carapace width (CCW) were measured according to Jensen *et al*. [18]. Individuals with a CCL of 35–65 cm are classified as small juveniles, subadults, and adults with a CCL of 66.0–86 cm [18].

### DNA extraction and polymerase chain reaction (PCR) amplification

Genomic DNA was extracted from the blood samples using the Omega Bio-tek^®^ Blood DNA Kit method (Omega Bio-Tek, Inc.; GA, USA). Initially, 950-bp fragments of the mtDNA control region were amplified using the following primers: LCM15382 (5’-GCT TAA CCC TAA AGC ATT GG-3’) and H950 (5’-GTC TCG GAT TTA GGG GTTTG-3’) [18]. The PCR mixture (100 μL) was composed of 10 μL of 10 × buffer (20 mM Tris-HCl (pH 8.4), 50 mM KCl_2_), 2 μL of 10 mM dNTPs, 5 μL of 50 mM MgCl_2_, 1 μL of 100 pmol of each of the forward and reverse primers, 0.5 μL of 5 units/μL of Taq DNA polymerase (Invitrogen™; CA, USA), 10 μL of DNA template, and distilled water to make the total volume 100 μL. After an initial denaturing at 94°C for 3 min, the amplification was performed using 35 cycles at 94°C for 30 s, annealing at 55°C for 30 s, and extension at 72°C for 60 s, with a final extension at 72°C for 3 min. The expected PCR products were approximately 950 bp in size. The PCR products were analyzed using 1% agarose gel electrophoresis at 100 V for 30 min and visualized under ultraviolet illumination. The PCR products were submitted for bidirectional sequencing at First BASE Laboratories Sdn Bhd (Selangor, Malaysia).

### Phylogenetic and haplotype network construction

We edited, assembled, and aligned the mtDNA control region sequences using the BioEdit software (version 7.1.3; Ibis Biosciences; Carlsbad, CA, USA). To confirm correctness, the alignment of each mtDNA control region sequence was checked by the eye. Subsequently, mtDNA control region sequences from Indo-Pacific MUs and rookeries [7, 19–21] and all available sequences published in GenBank database (https://www.ncbi.nlm.nih.gov/) were used to align and conduct a phylogeographic study to identify haplotypes and clades for the sequences obtained in the present study. According to Jensen *et al*. [7], 384-bp mtDNA control region sequences were used for the phylogenetic and clade analyses. Thus, the obtained 770 bp sequences were trimmed to 384 bp using the CmP nomenclature and clade according to Jensen *et al*. [7] for further comparison with available data. Phylogeographic relationships were inferred using MrBayes version 3.2.6 software (https://nbisweden.github.io/MrBayes/download.html) by constructing a neighbor-joining (NJ) tree, including the flatback turtle (*Natator depressus*) as an outgroup [22]. FigTree software version 1.4.3 (http://tree.bio.ed.ac.uk/software/figtree/) was used to view the tree. Templeton-Crandall-Sing (TCS) v. 1.21 [23] was used to estimate the most parsimonious genealogy between haplotypes, with the resulting networks constructed using PopART v1.1 beta software (http: www.leigh.net.nz/software.shtml) [24]. The number of haplotypes was based on the 384 bp sequence version; however, decimal suffixes (CmPx.1) were based on the equivalent longer version (770 bp) of the same haplotype. If similar haplotypes were not identified, the sequence was considered to be a new haplotype.

### Statistical analysis

The number of haplotypes, haplotype diversity (*h*), nucleotide diversity (π), and number of polymorphic sites were determined using the 770-bp sequences of the mtDNA control region based on DnaSP v.6 software [25]. Population differentiation among collection sites was evaluated using analysis of molecular variance (AMOVA; 10,000 permutations) [26]. The statistical significance of population genetic differentiation based on haplotype distribution across populations was examined using Fisher’s exact test in FSTAT 2.9.3 software [27] and was conducted with 500,000 steps in a Markov chain with 10,000 dememorization steps. Hierarchical AMOVA was performed to assess the proportions of genetic variation within and among populations using five packages implemented in R software (R Core Team, 2021) [28]. Both pairwise *F*_ST_ and Φ_ST_ (Kimura 2-parameter model) distance measures were used to calculate the levels of within- and among-population diversity based on 10,000 permutations using Arlequin v. 3.5.2.2 software [29]. In addition, these tests were conducted to compare samples from Gulf of Thailand and Indo-Pacific MUs and rookeries [7]. In general, population pairwise tests (Φ_ST_ or *F*_ST_) showed no or little genetic differentiation between rookeries located within 500 km of each other and significant structures between more distant rookeries; however, rookeries separated by more than 1,000 km were significantly differentiated from each other [7]. Therefore, UGT was combined with EGT due to the small number of stranded turtles (n = 3), where the distance between UGT and EGT was <500 km (approximately 200 km).

## Results

Of the 91 turtles, 48, 3, 21, and 19 green turtles were found at EGT, UGT, CGT, and LGT, respectively (Table-1). The means and ranges of CCL and CCW were 48.7 ± 20 cm (range, 20–100 cm) and 45.3 ± 19.6 cm (range, 19–92 cm), respectively, as shown in Table-1. Most of the green turtles were small juveniles (72/91; 79.12%), followed by subadults (11/91; 12.09%), and adults (8/91; 8.79%) based on CCL length. Small juvenile turtles were predominant at all collection sites except UGT (1/3).

**Table-1:** Collecting sites, numbers of green turtles, CCL, CCW, and size class of green turtles in this study.

Sites	Number	CCL (cm)	CCW (cm)	Small juvenile	Subadult	Adult
EGT	48	44.2 ± 19.3 (Range 20–87)	41.2 ± 19.7 (Range 42–82)	41	3	4
UGT	3	64.7 ± 18.9 (Range 38–80)	67.7 ± 18.2 (Range 20–91)	1	2	-
CGT	21	60.4 ± 20.1 (Range 31–100)	55.6 ± 18.0 (Range 29–92)	14	3	4
LGT	19	45.0 ± 14.8 (Range 24–87)	41.0 ± 13.9 (Range 22–69)	16	3	-
Total	91	48.7 ± 20 (Range 20–100)	45.3 ± 19.6 (Range 19–92)	72	11	8

EGT=Eastern Gulf of Thailand, UGT=Upper Gulf of Thailand, CGT=Central Gulf of Thailand, LGT=Lower Gulf of Thailand, CCl=Curved carapace length, CCW=Curved carapace len

**Figure-2:**
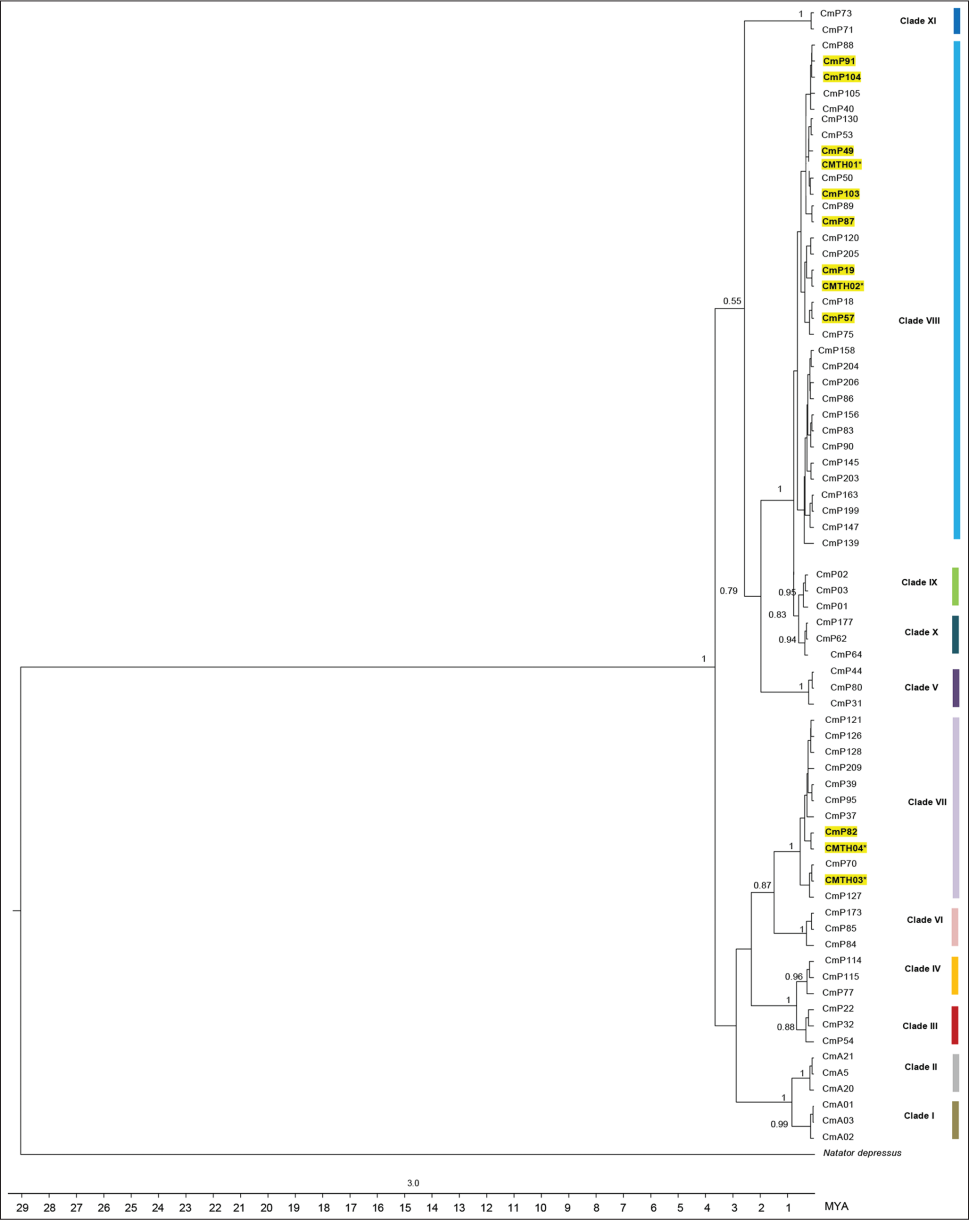
Phylogenetic tree of green turtles describing relationships among 71 mitochondrial DNA control region haplotypes (384 bp) from 11 clades [7], including haplotypes from UC-Eastern Gulf of Thailand and lower Gulf of Thailand of the present study. Tree shows percentage bootstrap support, with branch lengths proportional to the percentage sequence divergence indicated by the scale. Yellow block and asterisk (*) indicate known haplotypes and new haplotypes identified in this study, respectively. *Natator depressus* was the outgroup for this analysis.

**Figure-3:**
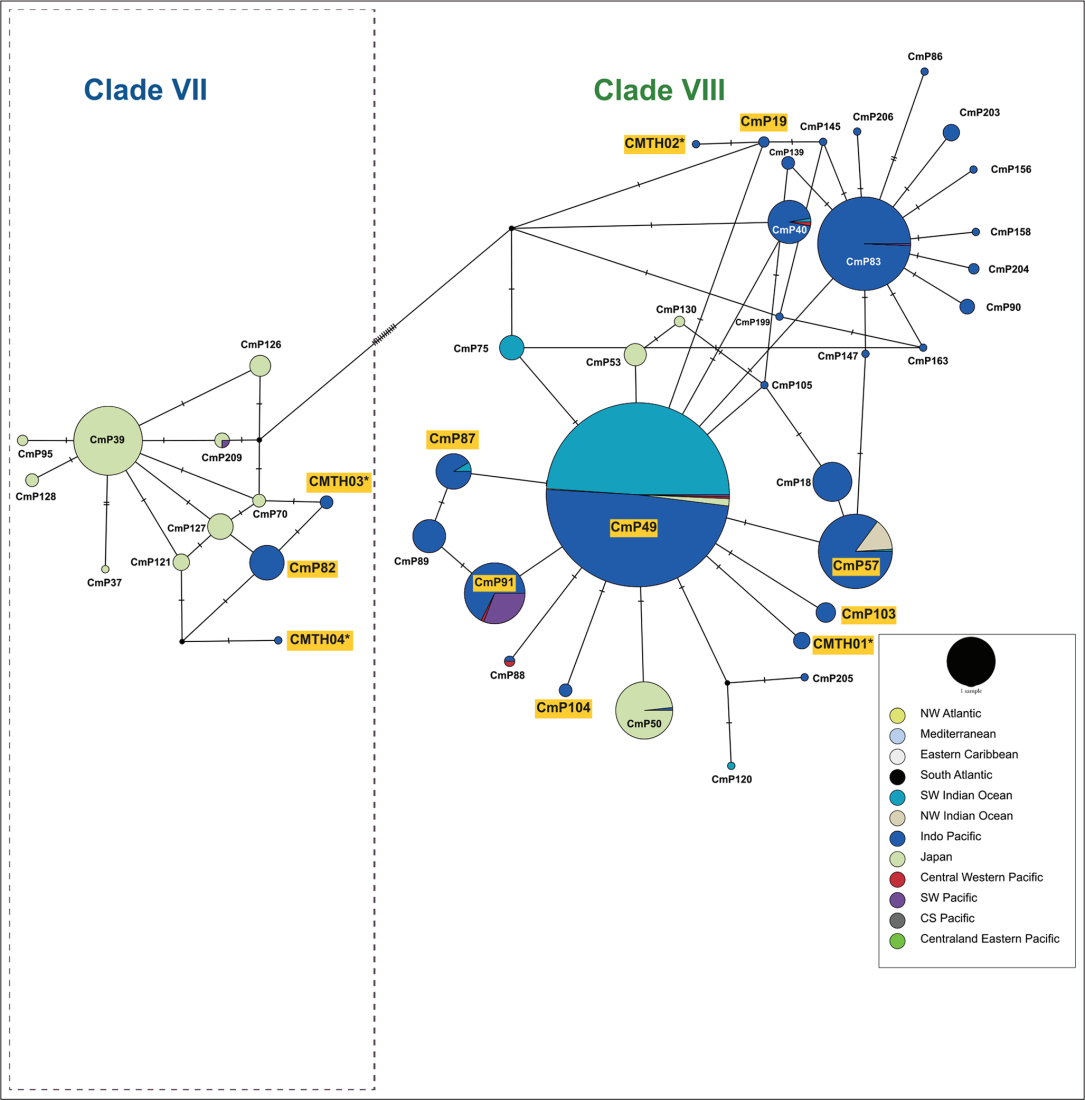
Haplotype network diagram (based on 384 bp) showing relationships between 45 haplotypes from clades VII and VIII, including 12 haplotypes found in this study. Number of mutations illustrated by dashes in connecting lines and missing intermediates haplotypes represented by black circles. Yellow block and asterisk (*) indicate known haplotypes and new haplotypes identified in this study, respectively. The size of pie charts indicates relative frequency of each haplotype and colors in pie diagram represent the 12 geographic groups (NWATL=NW Atlantic, ECARIB=Eastern Caribbean, MED=Mediterranean, SATL=South Atlantic, SWIO=SW Indian Ocean, NWIO=NW Indian Ocean, IP=Indo-Pacific, JP=Japan, CWP=Central West Pacific, SWP=SW Pacific, CSP=Central South Pacific, and C&EP=Central and Eastern Pacific).

There were 33 polymorphic sites corresponding to 32 transitions and one transversion on the 770-bp sequences (Table-2). Twelve haplotypes and two clades were identified for 384-bp fragments (Figure-1). CmP49, CmP57, CmP87, CmP91, CmP103, CmP104, CMTH01, and CMTH02 were classified as clade VIII, whereas CMP82, CMTH03, and CMTH04 were classified as clade VII (Figure-2 and Table-3) [7]. Four new variant haplotypes (CMTH01, CMTH02, CMTH03, and CMTH04; accession numbers OR247781–OR247784) were identified in the present study (Table-2). The 13^th^ haplotype (CMTH05; accession number OR247785) belonging to clade VIII was identified on the basis of the 770-bp fragments (Table-3). The mtDNA control region haplotype network divided the sampled green turtles in the Gulf of Thailand into two clusters (Clades VII and VIII), which was consistent with the phylogenetic tree (Figure-3). Four out of 45 CmP49.1 were identified as a new variant haplotype (CMTH05) due to a G-to-A transition at nucleotide 635 according to the 770 bp sequence. Furthermore, CMTH01 had an A-to-G transition from CmP49 at nucleotide 77, and CMTH02 also had an A-to-G transition from CmP19 at nucleotide 120 (Table-2 and Figure-3). CMTH03 differed from CmP82 by a G-to-A transition at nucleotide 253. According to the mtDNA control region networks, CMTH04 should have a mutation from the missing intermediate haplotype (Figure-3) and had two T-to-C transitions from CmP82 at nucleotides 221 and 320. CMTH03 and CMTH04 differed by 3 transitions (A-to-G at nucleotide 101 and 2 T-to-C transitions at nucleotides 221 and 320, respectively). The two most common haplotypes for the Gulf of Thailand were CmP49 (45/91; 49.45%) and CmP82 (17/91; 18.68%). CmP49.1 was also the most common haplotype for the Gulf of Thailand (41/91; 45.05%); however, CmP82 was found only at U-EGT and CGT. All other haplotypes were relatively rare (<7% each). For the new haplotype, CMTH05 was found at all collection sites (U-EGT (n = 1), CGT (n = 2), and LGT (n = 1)) (4/91; 4.39%). CMTH01 and CMTH03 were found only at U-EGT (8/91; 8.79%), whereas CMTH02 and CMTH04 were found only at CGT (2/91; 2.20%). A single CmP19 was found only in the LGT. The haplotype network diagrams (Figure-3) reflect the evolutionary relationships among the haplotypes of green turtles in the Gulf of Thailand.

**Table-2:** Polymorphic sites (384 bp fragment) defining 12 haplotypes, numbers of haplotypes, number per collection site, and haplotype frequency.

Variable sites in sequences 384 bp																											Locality			% Haplotype
	
Haplotype	46	57	61	62	63	77	78	79	101	120	154	159	185	221	245	253	259	261	273	298	299	320	326	337	358	N	U-EGT	CGT	LGT	
CmP49	G	G	C	C	A	A	A	T	A	A	A	C	G	T	G	T	A	A	C	C	T	T	C	T	A	45	26	5	14	49.45%
CmP104	.	.	.	.	.	.	.	.	.	.	.	.	.	.	.	.	.	.	.	.	.	.	.	.	C	2	1	0	1	2.19%
CmP87	.	.	.	.	.	.	.	C	.	.	.	.	.	.	.	.	.	.	.	.	.	.	.	.	.	2	1	1	0	2.19%
CmP57	.	.	.	.	.	.	G	.	.	.	.	.	.	.	.	.	.	.	.	.	.	.	.	.	.	4	3	1	0	4.39%
CmP103	.	.	.	.	.	.	.	.	.	.	G	.	.	.	.	.	.	.	.	.	.	.	.	.	.	4	1	1	2	4.39%
CmP91	.	.	.	.	.	.	.	.	.	.	.	.	.	.	A	.	.	.	.	.	.	.	.	.	.	6	2	3	1	6.59%
CmP19	.	.	.	.	.	.	.	.	.	.	.	.	A	.	.	.	.	.	.	.	.	.	.	.	.	1	0	0	1	1.09%
CmP82	A	A	T	T	G	.	.	.	G	.	.	T	A	.	A	C	G	G	T	T	C	.	T	C	.	17	9	8	0	18.68%
CMTH01	.	.	.	.	.	G	.	.	.	.	.	.	.	.	.	.	.	.	.	.	.	.	.	.	.	5	5	0	0	5.49%
CMTH02	.	.	.	.	.	.	.	.	.	G	.	.	A	.	.	.	.	.	.	.	.	.	.	.	.	1	0	1	0	1.09%
CMTH03	A	A	T	T	G	.	.	.	.	.	.	T	A	.	A	C	G	G	T	T	C	.	T	C	.	3	3	0	0	3.29%
CMTH04	A	A	T	T	G	.	.	.	G	.	.	T	A	C	A	C	G	G	T	T	C	C	T	C	.	1	0	1	0	1.09%

EGT=Eastern Gulf of Thailand, CGT=Central Gulf of Thailand, LGT=Lower Gulf of Thailand, Dot means the same base according to the reference haplotype (the uppermost haplotype in the table) at the same position. It is the universal sign to use to indicate the same base

**Table-3:** Collection sites, numbers of turtles, haplotype, clade, nucleotide diversity (π), and haplotype diversity (*h*) of 13 haplotypes (770 bp sequences), where clades used follow Jensen *et al.* [7].

Sampling site	Number (N)	Haplotype number	Haplotype	Clade	Nucleotide diversity (p)	Haplotype diversity (*h*)
U-EGT	51	10	CmP49.1 CmP57.1 CmP87.1 CmP82.1 CmP91.1 CmP103.1 CmP104.1 CmTH01 CmTH03 CmTH05*	VIII VIII VIII VII VIII VIII VIII VIII VII VIII	0.01155 ± 0.00278	0.723 ± 0.057
CGT	21	9	CmP49.1 CmP57.1 CmP82.1 CmP87.1 CmP91.1 CmP103.1 CmTH02 CmTH04 CmTH05*	VIII VIII VII VIII VIII VIII VIII VII VIII	0.01633 ± 0.00413	0.833 ± 0.066
LGT	19	6	CmP49.1 CmP104.1 CmP103.1 CmP91.1 CmP19.1 CmTH05*	VIII VIII VIII VIII VIII VIII	0.00080 ± 0.001	0.538 ± 0.133
Total	91	13			0.01146 ± 0.00248	0.755 ± 0.039

* CMTH05 (based on 384 bp) cannot be distinguished from CmP49 and belongs to clade VIII due to phylogenetic tree based on 770 bp (data not shown). EGT=Eastern Gulf of Thailand, CGT=Central Gulf of Thailand, LGT=Lower Gulf of Thailand

The overall haplotype and nucleotide diversity of green turtles from the Gulf of Thailand were 0.755 ± 0.039 and 0.01146 ± 0.00248, respectively (Table-3). Central Gulf of Thailand (*h* = 0.833 ± 0.066 and π = 0.01633 ± 0.00413) and LGT (*h* = 0.538 ± 0.133 and π = 0.00080 ± 0.001) had the highest and lowest haplotypes and nucleotide diversity, respectively. Western New Caledonia had higher haplotype and nucleotide diversity than the Gulf of Thailand (Table-4) [8, 21, 22, 30–32]. Brunei Bay, Sangalaki, and Eastern Borneo have higher haplotype diversity than the Gulf of Thailand. Wanan has higher nucleotide diversity than the Gulf of Thailand. The genetic variability within populations using AMOVA was 85.74% for the three collection sites. The variance component among populations was 14.26%, which was smaller than within populations (*F*_ST_ = 0.14258, p = 0.00293), but was statistically significant. A significant difference in pairwise *F*_ST_ (*F*_ST_ = 0.12874; p = 0.02703) was observed between U-EGT and LGT (Table-5a). In addition, CGT population was significantly different from LGT population (*F*_ST_ = 0.36740; p = 0.00001). However, no significant difference was observed between U-EGT and CGT (*F*_ST_ = 0.06397; p = 0.09009). Exact tests confirmed population differentiation at all collection sites. The *F*_ST_ results indicated that U-EGT and CGT were combined (UC-EGT) because there was no significant difference between them. Subsequently, population subdivision between UC-EGT and LGT was tested. Comparing the two collection sites (UC-EGT and LGT), AMOVA revealed 83.48% genetic variability within populations and 16.52% variance among populations (*F*_ST_ = 0.16523, p = 0.00978). UC-EGT significantly differed from LGT (*F*_ST_ = 0.16655, p = 0.00901; exact test, p = 0.04164) (Table-5b). Furthermore, Φ_ST_ was in accordance with *F*_ST_ for comparisons between the three sites or between the two sites (data not shown).

**Table-4:** Comparison of genetic diversity of green turtles (384 bp) from Gulf of Thailand and other locations in Western Pacific.

Region	Management unit	Haplotype number	Haplotype diversity (h) ± SD	Nucleotide diversity (p) ± SD	Number of samples
South China Sea	Gulf of Thailand	12	0.755 ± 0.04	0.012 ± 0.00	91
	U-EGT	10	0.723 ± 0.06	0.012 ± 0.00	51
	CGT	9	70.833 ± 0.0	0.016 ± 0.00	21
	LGT	6	0.538 ± 0.13	± 0.0010.00	19
	Peninsular Malaysia^A^	8	0.645 ± 0.09	0.008 ± 0.00	29
	Sipadan, Malaysia^A^	7	0.630 ± 0.07	0.005 ± 0.00	98
	Sarawak, Malaysia^A^	3	0.450 ± 0.11	0.009 ± 0.00	22
	Western Borneo^A^	3	0.450 ± 0.11	0.009 ± 0.01	22
	Brunei Bay^B^	10	0.812 ± 0.04	0.012 ± 0.01	42
	Paracel Islands^C^	12	0.415 ± 0.07	0.002 ± 0.00	72
	Hainan^D^	10	0.721 ± 0.04	0.005 ± 0.00	85
Arafura Sea	Aru^A^	2	0.071 ± 0.07	0.004 ± 0.00	28
	Gulf of Carpentaria^A^	7	0.659 ± 0.05	0.011 ± 0.01	50
	Cobourg Peninsula^A^	5	0.573 ± 0.08	0.002 ± 0.00	37
Sulu Sea	Sulu Sea^A^	3	0.323 ± 0.07	0.001 ± 0.00	62
East Indian Ocean	West Java^A^	3	0.515 ± 0.08	0.001 ± 0.00	22
	Cocos Keeling Island^A^	2	0.199 ± 0.11	0.007 ± 0.01	19
Celebes Sea	Eastern Borneo^A^	5	0.763 ± 0.04	0.007 ± 0.00	29
	Sangalaki^A^	5	0.780 ± 0.16	0.008 ± 0.03	92
	North East Borneo^A^	7	0.633 ± 0.04	0.004 ± 0.00	
Timor Sea	Ashmore Reef^A^	5	0.632 ± 0.04	0.005 ± 0.00	44
	Scott Reef/Brown Island^A^	4	0.510 ± 0.06	0.001 ± 0.00	65
Japan	Ogasawa^E^	13	0.706 ± 0.04	0.016 ± 0.01	103
Taiwan	Wanan^F^	3	0.483 ± 0.06	0.028 ± 0.01	40
	Lanyu^F^	1	0.000 ± 0.00	0.000 ± 0.00	14
South-west Pacific Ocean	North Great Barrier Reef^A^	10	0.632 ± 0.06	0.012 ± 0.00	81
	Western New Caledonia^A^	11	0.817 ± 0.02	0.031 ± 0.02	64
	French Polynesia^A^	2	0.222 ± 0.17	0.000 ± 0.00	9
North-west Pacific Ocean	Northern New Guinea^A^	3	0.216 ± 0.12	0.012 ± 0.01	18
	Palau^A^	2	0.056 ± 0.05	0.002 ± 0.00	36
	Guam^A^	2	0.042 ± 0.04	0.000 ± 0.00	47

A Jensen *et al.* [8],

B Joseph *et al.* [30],

C Li *et al.* [22],

D Gaillard *et al.* [21],

E Nishizawa *et al.* [31],

F Cheng *et al.* [32]. EGT=Eastern Gulf of Thailand, CGT=Central Gulf of Thailand, LGT=Lower Gulf of Thailand, SD=Standard deviation

**Table-5:** Genetic population differentiation (AMOVA) based on mtDNA haplotypes (770 bp) (a, b). Statistical analysis among U-EGT, CGT and LGT (a) and between UC-EGT (combined between U-EGT and CGT) and LGT (b). Above diagonal shows *P* values of exact tests and below diagonal shows *F*_ST_ (a, b).

(a) mtDNA-based population differentiation (among three collection sites)			

Collection site	U-EGT	CGT	LGT
U-EGT	-	**p = 0.01822**	**p = 0.04746**
CGT	0.06397 (p = 0.09009)	**-**	**p = 0.00072**
LGT	**0.12874 (p = 0.02703)**	**0.36740 (p = 0.0000)**	-

AMOVA=Analysis of molecular variance, mtDNA=mitochondrial DNA, EGT=Eastern Gulf of Thailand, CGT=Central Gulf of Thailand, LGT=Lower Gulf of Thailand, Bold values indicate significant differentiation and *P* values of permutation tests (p < 0.05)

**Table:** 

(b) mtDNA-based population differentiation (between two collection sites)		

Collection site	UC-EGT	LGT
UC-EGT	-	**p = 0.04164**
LGT	**0.16655 (p = 0.00901)**	-

mtDNA=mitochondrial DNA, EGT=Eastern Gulf of Thailand, CGT=Central Gulf of Thailand, LGT=Lower Gulf of Thailand, Bold values indicate significant differentiation and *P* values of permutation tests (p < 0.05)

Population differentiation between UC-EGT or LGT and Indo-Pacific MUs and rookeries was confirmed by exact tests, *F*_ST_, and Φ_ST_ (Table-6) [8, 12, 30–35]. The exact tests showed significant differentiation between the UC-EGT and all the MUs and rookeries, with the exception of the Eastern Taiwan (Lanyu) MU and the Vietnam rookery and between the LGT and all the MUs and rookeries, with the exception of the Peninsular Malaysia, Eastern Taiwan (Lanyu), and Cocos Keeling Island MUs and the Perak, Vietnam, and Terengganu rookeries (Table-6) [8, 12, 30–35]. *F*_ST_ values for comparison between UC-EGT and all MUs and rookeries ranged from 0.07764 to 0.76540, with the exception of the Mersing rookery, showing significant differentiation. Comparing LGT with all the MUs and rookeries, the *F*_ST_ values were in the range 0–0.95994, with all the MUs and rookeries showing significant differentiation, with the exception of the Peninsular Malaysia, Sipadan, Brunei Bay, Eastern Taiwan (Lanyu), Scott Reef and Browse Island, and Cocos Keeling Island MUs and the Perak, Redang, and Vietnam rookeries. For UC-EGT, the Φ_ST_ test showed significant differentiation with all MUs and rookeries, with the exception of Peninsular Malaysia and Eastern Taiwan (Lanyu) MUs and Terrangganu and Mersing. The Φ_ST_ test also showed significant differentiation between LGT and all MUs and rookeries, with the exception of Peninsular Malaysia, Sipadan, Brunei Bay, Eastern Taiwan (Lanyu), Scott Reef and Browse Island, and Gulf of Carpentaria MUs and Perak, Perhentain Island, Redang, Pahang, and Vietnam rookeries.

**Table-6:** Genetic differentiation values of exact test, *F*_ST_, and Φ_ST_ of green turtles from Indo-Pacific MUs and rookeries (*italics*) and green turtles from UC-EGT or LGT (p<0.5 to infer significance).

Region	MU	Country	No.	UC-EGT			LGT		
	
				Exact test (p-value)	*F*_ST_	F_ST_	Exact test (p-value)	*F*_ST_	*Φ*_ST_
Southeast Asia	Peninsula Malaysia^A^	Malaysia	29	p (0.0407)	0.100, P (0.0180)	0.102, P (0.0540)	p (0.8892)	−0.003, P (0.5045)	−0.004, P (0.4594)
	Sipadan^B^	Malaysia	103	p (<0.0001)	0.169, P (<0.0001)	0.152, P (<0.0001)	p (0.0007)	0.028, P (0.0720)	0.017, P (0.0991)
	Sarawak^B^	Malaysia	149	p (<0.0001)	0.329, P (<0.0001)	0.315, P (<0.0001)	p (<0.0001)	0.329, P (<0.0001)	0.409, P (<0.0001)
	Sulu sea^B^	Malaysia/Philippines	62	p (<0.0001)	0.306, P (<0.0001)	0.324, P (<0.0001)	p (<0.0001)	0.579, P (<0.0001)	0.630, P (<0.0001)
	Berau^B^	Indonesia	29	p (<0.0001)	0.143, P (0.0090)	0.132, P (0.0090)	p (0.0002)	0.064, P (0.0090)	0.061, P (0.02703)
	Eastern Borneo^B^	Indonesia	29	p (<0.0001)	0.143, P (0.0090)	0.132, P (0.0090)	p (<0.0001)	0.062, P (<0.0001)	0.058, P (0.0090)
	Western Borneo^B^	Indonesia	22	p (<0.0001)	0.121, P (<0.0001)	0.130, P (0.0180)	p (<0.0001)	0.183, P (<0.0001)	0.159, P (<0.0001)
	Aru^B^	Indonesia	28	p (<0.0001)	0.182, P (<0.0001)	0.153, P (<0.0001)	p (<0.0001)	0.360, P (<0.0001)	0.368, P (<0.0001)
	New Guinea^B^	Papua New Guinea	18	p (<0.0001)	0.648, P (<0.0001)	0.672, P (<0.0001)	p (<0.0001)	0.870, P (<0.0001)	0.882, P (<0.0001)
	Brunei Bay^D^	Brunei	42	p (<0.0001)	0.104, P (<0.0001)	0.090, P (<0.0001)	p (<0.0001)	0.042, P (0.10811)	0.030, P (0.0991)
	Eastern Taiwan (Lanyu)^E^	Taiwan	14	p (0.1400)	0.158, P (0.0180)	0.156, P (0.0720)	p (0.5967)	−0.005, P (0.6936)	0.003, P (0.5045)
	Western Taiwan (Wanan)^E^	Taiwan	40	p (<0.0001)	0.270, P (<0.0001)	0.251, P (<0.0001)	p (<0.0001)	0.448, P (<0.0001)	0.236, P (<0.0001)
	Paracell^F^	China	16	p (<0.0001)	0.119, P (0.0540)	0.089, P (0.0270)	p (<0.0001)	0.163, P (<0.0001)	0.145, P (<0.0001)
Northwest Pacific	Guam^G^	Guam	47	p (<0.0001)	0.765, P (<0.0001)	0.785, P (<0.0001)	p (<0.0001)	0.959, P (<0.0001)	0.964, P (<0.0001)
	Palau^B^	Palau	36	p (<0.0001)	0.751, P (<0.0001)	0.778, P (<0.0001)	p (<0.0001)	0.959, P (<0.0001)	0.973, P (<0.0001)
Indian Ocean	Cocos Keeling Island^B^	Australia	19	P (0.0065)	0.095, P (0.0450)	0.078, P (0.0270)	p (0.0783)	0.043, P (0.3873)	0.044, P (0.3243)
	Western Java^B^	Indonesia	22	p (<0.0001)	0.168, P (<0.0001)	0.160, P (<0.0001)	p (0.0065)	0.105, P (0.0090)	0.143, P (<0.0001)
East Indian Ocean	Scott Reef and Browse Island^B^	Australia	65	p (<0.0001)	0.206, P (<0.0001)	0.202, P (<0.0001)	p (0.0003)	0.039, P (0.0630)	0.013, P (0.07207)
	Ashmore Reef^B^	Australia	44	p (<0.0001)	0.222, P (<0.0001)	0.203, P (<0.0001)	p (<0.0001)	0.337, P (<0.0001)	0.335, P (<0.0001)
	Gulf of Carpentaria^B^	Australia	49	p (<0.0001)	0.150, P (<0.0001)	0.136, P (<0.0001)	p (<0.0001)	0.073, P (0.0450)	0.043, P (0.0630)
	Cobourg Peninsula^B^	Australia	37	p (<0.0001)	0.247, P (<0.0001)	0.257, P (<0.0001)	p (<0.0001)	0.405, P (<0.0001)	0.438, P (<0.0001)
Coral Sea	NGBR^B^	Australia	47	p (<0.0001)	0.574, P (<0.0001)	0.572, P (<0.0001)	p (<0.0001)	0.799, P (<0.0001)	0.783, P (<0.0001)
Southwest Pacific	French Polynesia^G^	Australia	9	p (<0.0001)	0.600, P (<0.0001)	0.678, P (<0.0001)	p (<0.0001)	0.961, P (<0.0001)	0.985, P (<0.0001)
Southeast Asia	*Perak* ^C^	Malaysia	19	p (0.0072)	0.168, P (0.0270)	0.164, P (0.0180)	p (0.2502)	0.023, P (0.11712)	0.028, P (0.1351)
	*Perhentian Islands* ^C^	Malaysia	22	p (<0.0001)	0.211, P (<0.0001)	0.184, P (<0.0001)	p (0.0208)	0.132, P (0.0450)	0.084, P (0.0630)
	*Redang* ^C^	Malaysia	134	p (<0.0001)	0.118, P (<0.0001)	0.121, P (<0.0001)	p (0.0297)	0.014, P (0.2162)	0.016, P (0.2342)
	*Terengganu* ^C^	Malaysia	12	p (0.0322)	0.077, P (0.0360)	0.044, P (0.1801)	p (0.1176)	0.069, P (0.0360)	0.075, P (0.0090)
	*Pahang* ^C^	Malaysia	27	p (<0.0001)	0.182, P (<0.0001)	0.205, P (<0.0001)	p (0.0021)	0.198, P (0.0180)	0.009, P (0.3603)
	*Mersing* ^C^	Malaysia	7	p (0.0084)	0.127, P (0.0630)	0.104, P (0.1441)	p (0.0016)	0.436, P (<0.0001)	0.579, P (<0.0001)
	*TIP* ^C^	Malaysia	178	p (<0.0001)	0.462, P (<0.0001)	0.483, P (<0.0001)	p (<0.0001)	0.716, P (<0.0001)	0.740, P (<0.0001)
	*Penang* ^C^	Malaysia	22	p (0.0073)	0.182, P (0.0090)	0.180, P (<0.0001)	p (0.0061)	0.021, P (0.00901)	0.032, P (<0.0001)
	*Veitnam* ^C^	Veitnam	14	p (0.09231)	0.158, P (<0.0001)	0.156, P (0.0360)	p (0.2352)	−0.005, P (0.7387)	0.003, P (0.4774)
Northwest Pacific	*Southwest. Irriomote* ^H^	Japan	26	p (<0.0001)	0.358, P (<0.0001)	0.340, P (<0.0001)	p (<0.0001)	0.514, P (<0.0001)	0.499, P (<0.0001)
	*Eastern Ishigaki* ^H^	Japan	29	p (<0.0001)	0.600, P (<0.0001)	0.623, P (<0.0001)	p (<0.0001)	0.784, P (<0.0001)	0.809, P (<0.0001)
	*Northwest. Ishigaki* ^H^	Japan	12	p (<0.0001)	0.568, P (<0.0001)	0.602, P (<0.0001)	p (<0.0001)	0.827, P (<0.0001)	0.867, P (<0.0001)

A Dethmers *et al.* [34],

B Jensen *et al.* [8],

C Nishizawa *et al.* [35]**,**

D Joseph *et al.* [30],

E Cheng *et al.* [32],

F Song *et al.* [12],

G Dutton *et al.* [33],

H Nishizawa *et al.* [31]. EGT=Eastern Gulf of Thailand, LGT=Lower Gulf of Thailand, MUs=Management units

## Discussion

For the first time, this study reports the haplotypes and clades of green turtles in the Gulf of Thailand. Twelve haplotypes (based on 384 bp) or 13 haplotypes (based on 770 bp) were identified and distributed into two clades (VII and VIII) in this study. Four or five new haplotypes based on 384 bp or 770 bp, respectively, were identified. For 384 bp of the mtDNA control region sequences, clade VIII consisted of CmP19.1, CmP49, CmP57, CmP87, CmP91, CmP103, CmP104, CMTH01, and CMTH02, whereas clade VII consisted of CmP82, CMTH03, and CMTH04. CMTH05 was separated from CmP49.1 with a G-to-A transition at nucleotide 627 for 770 bp of the mtDNA control region sequences. This result is in accordance with previous studies by Jensen *et al*. [7], Dutton *et al*. [36], and Shamblin *et al*. [37] those showed that longer mtDNA fragments (770 bp) could increase the resolution of the stock structure in some regions.

All haplotypes of clade VIII in the present study were commonly observed in South-east Asian MUs [7]. CmP49 is a common haplotype in the South-west Pacific MU [7], Micronesian MU [33], Eastern Indian Ocean rookeries [7], and Ryukus rookeries in Japan [38]. In addition, CmP91 and CmP49 have been found in these MUs and rookeries, with the exception of the Ryukus rookeries in Japan. CmP103 has been found only on Peninsular Malaysia [7]. CMTH01 and CMTH02 haplotypes were derived from mutations of CmP49 and CmP19, respectively. All five CMTH01 and one CMTH02 samples were collected from U-EGT and CGT, respectively, which may be specific to these collection sites.

Three haplotypes of clade VII (17 CmP82, three CMTH03, and one CMTH04) were found at U-EGT and CGT but not at LGT in this study. According to Jensen *et al*. [7], and Hamabata *et al*. [38], clade VII with nine haplotypes—CmP37 (n = 1), CmP39 (n = 87), CmP70 (n = 3), CmP95 (n = 2), CmP121 (n = 5), CmP126 (n = 8), CmP127 (n = 12), CmP128 (n = 3), and CmP209 (n = 3)—is unique in the North-west (NW) Pacific rookeries in Japan, with The present study identified 17 CmP82 samples, with other studies identifying 18 CmP82 samples in the Malaysia and Brunei MUs and rookeries (Peninsular Malaysia (n = 2), Brunei Bay (n = 1), West Borneo, Indonesian (n = 1), and Sipadan (n = 1) MUs and in the Redang (n = 12), and Terrengganu (n =1) rookeries) [7, 8, 30, 34, 35]. In combination with the present study, these reports suggest that CmP82 should not be considered as a low-frequency haplotype. Two new haplotypes of clade VII were found only in the present study. Hamabata *et al*. [38], suggested that CmP39 was the ancestral haplotype of clade VII, which has been a surviving lineage in the NW Pacific rookeries and has extended from the NW Pacific to South-east Asia by gene flow resulting from incidental nest site shifts to the Sunda Shelf (Peninsular Malaysia and Sarawak). The haplotype network diagram developed in the present study shows that CmP82 branching from CmP127 and CmP127 branching from CmP39. In the present study, two new haplotypes (CMTH03 and CMTH04) of clade VII also branch from CmP82, and an intermediate haplotype branching from CmP82 is missing. These results are in agreement with those reported by Hamabata *et al*. [38], who reported that CmP82, CMTH03, and CMTH04 may have resulted from the evolutionary process of CmP39. Nevertheless, clade VII is unique not only in the NW Pacific (Japan) but also in Thailand and Malaysia. In addition, CmP82 should be considered as a unique haplotype of the Gulf of Thailand and Malaysia. According to the results of the present study, clade VII should have 12 haplotypes now.

Overall, the haplotype diversity of green turtles in the Gulf of Thailand was high (0.755 ± 0.04) and higher than that of most of the MUs in the South China Sea, with the exception of Brunei Bay (0.812 ± 0.04), Eastern Borneo (0.763 ± 0.04), Sangalaki (0.780 ± 0.16) from the Celebes Sea, and Western New Caledonia (0.817 ± 0.02) in the South-west Pacific Ocean. The haplotype diversity of green turtles in the Gulf of Thailand was lower than the average haplotype diversity of the South-east Asian MUs (*h* = 0.80) but higher than the average haplotype diversity for the Pacific Ocean (*h* = 0.71) and Indian Ocean (*h* = 0.70) [34]. The nucleotide diversity of green turtles in the Gulf of Thailand was high (π = 0.012 ± 0.00) and higher than for most of the MUs in the South China Sea, with the exception of Wanan Island (π = 0.028 ± 0.01) and of Western New Caledonia (π = 0.031 ± 0.02) in the South-west Pacific Ocean. This nucleotide diversity value was higher than the average for the South-east Asian MUs (π = 0.006) but lower than the average for the Pacific Ocean (π = 0.34) and Indian Ocean (π = 0.19) [34]. The average haplotype and nucleotide diversities of the Gulf of Thailand were lower than the average for the Australasia MUs (*h* = 0.88; π = 0.040). In addition, the haplotype diversity of the Gulf of Thailand was lower than for the Atlantic populations (*h* = 0.83), though the nucleotide diversity was higher (π = 0.005) [34]. The haplotype and nucleotide diversities of the green turtles from the Gulf of Thailand were higher than the average global haplotype and nucleotide diversities (*h* = 0.420; π = 0.0093) [7]. Western New Caledonia had higher values for both haplotype and nucleotide diversities compared to the Gulf of Thailand, perhaps due to clade differences and the greater number of clades (four clades = VI, V, VI, and VIII) than for green turtles in the Gulf of Thailand (two clades = VII and VIII). Brunei Bay (clades III, VII, and VIII), Sangalaki (clades III and VIII), Eastern Borneo (clades III and VIII), and Wanan, Western Taiwan (clades III and VIII) had higher haplotype or nucleotide diversity than the Gulf of Thailand, which may have been due to clade differences, especially for clade III in this study.

The AMOVA indicated that green turtles in the Gulf of Thailand could be divided into two subpopulations (UC-EGT and LGT). Thus, UC-EGT and LGT were compared with Indo-Pacific MUs and rookeries. Notably, Φ_ST_ weighs more on genetic distance than haplotypic frequency (*F*_ST_), although both estimates explain most of the variation derived from inter-oceanic differences. However, *F*_ST_ is likely to be more affected by sample size; thus, Φ_ST_ is a more realistic value of inter-oceanic differentiation [39]. All the MUs used to compare with either UC-EGT or LGT in the present study that had a significant p-value for *F*_ST_ but a p-value for ΦST that was not significant had small sample sizes (Supplement-1). In the present study, Φ_ST_ was used to interpret the genetic differentiation of UC-EGT or LGT and Indo-Pacific MUs and rookeries. According to the Φ_ST_ analysis, UC-EGT was distinct from all collection sites except Peninsular Malaysia and Eastern Taiwan (Lanyu) MUs and Terangganu and Mersing rookeries. Based on the Φ_ST_ analysis, LGT was distinct from all collection sites with the exception of Peninsular Malaysia, Brunei Bay, Sipadan, Eastern Taiwan (Lanyu), Scott Reef and Browse Island, Cocos Keeling Island, Gulf of Carpentaria MUs and Perak, Perhentian Islands, Redang, Pahang, and Vietnam rookeries. Without any migration confirmation from satellite telemetry or tagging, it was not possible to confirm the presence of gene flow among the above MUs [21]. A satellite telemetry study on the migratory routes of green turtles in the Gulf of Thailand [15] reported that 11 green turtles released from Ko Kram in the Gulf of Thailand migrated to Peninsular Malaysia, the Sulu Sea, the coast of Brunei, Rong Island (Cambodia), and Phu Quy Island (Vietnam). Two green turtles traveled to Peninsular Malaysia (approximately 1260–1307 km) in 31–48 days. Three green turtles traveled to the Sulu Sea through North Borneo Island (approximately 2340–2823 km) in 43–45 days. One green turtle traveled to Brunei Island (approximately 2004 km) in 49 days, to Rong Island, Cambodia (approximately 456 km) in 16 days, and to Phu Quy Island via Cambodian waters (approximately 1263 km) in 19 days. According to satellite telemetry information, gene flow of green turtles would be possible from the Gulf of Thailand to the Malaysian and Brunei MUs and Vietnamese rookeries. These turtles may have migrated to the Sulu Sea and Brunei Bay due to foraging grounds in the Balabac Straits (between Borneo and the Philippines). Brunei Bay has recently been identified as a foraging ground for Asian green turtles [8, 30]. The CmP49 haplotype has been reported throughout the nesting and foraging grounds of Asia [8]. In the present study, CmP 49 was found in all MUs that were not genetically distinct to UC-EGT or LGT. Analytical power could be reduced by the widespread presence of shared, common haplotypes, many potential source MUs [40], relatively small sample sizes of both nesting and foraging populations, novel or orphan haplotypes [34], and incomplete sampling of potential nesting populations [20, 40]. Gene flow between the green turtles in the Gulf of Thailand and these MUs (Eastern Taiwan [Lanyu], Scott Reef and Browse Island, Cocos Keeling Island, and the Gulf of Carpentaria) could not be identified due to the lack of satellite telemetry information. The lack of genetic differentiation between these MUs and UC-EGT or LGT may be due to the shared common haplotype (CmP49). In conclusion, this report is the first to identify the genetic diversity, haplotypes, and clades of green turtles in the Gulf of Thailand. This study provides much-needed baseline genetic information which has increased our understanding of green turtle conservation in the Gulf of Thailand. The genetic analysis of green turtles in this study provides essential information regarding the green turtle stock structure in the Gulf of Thailand, which is crucial for the diagnosis, management, and monitoring of green turtle populations in the Gulf of Thailand. Clade VII was a unique clade not only for Japan but also for Thailand and Malaysia. In addition, CmP82 is a unique haplotype in the Gulf of Thailand and Malaysia. Cmp82, CMTH03, and CMTH04 may have a high priority for conservation due to the unique haplotypes specific to these regions. Based on size class, 71 out of 91 (79.12%) of the green turtles in the present study were small juveniles that could migrate long distances using oceanic currents in combination with active swimming periods [30]. The current results indicate that collaboration within South-east Asian countries (Thailand, Cambodia, Vietnam, Malaysia, Indonesia, and Brunei Darussalam) will be required because the migratory routes of green turtles from the Gulf of Thailand are similar. In addition, international cooperation with Taiwan and Australia will be necessary due to the shared common haplotypes (CmP49 and CmP91). The current results show that the populations in the Gulf of Thailand have high genetic diversity and haplotypic endemism. Conservation and management of these populations are important to preserve genetic diversity and biological diversity and evolutionary potential of this species. Widespread shared haplotypes hinder the identification of the origin of several haplotypes endemic to the Gulf of Thailand. Therefore, future studies will focus on male-mediated gene flow using nuclear DNA microsatellite or single-nucleotide polymorphisms to further improve our understanding of the demographic connectivity among populations.

## Conclusion

Thirteen haplotypes of green turtles in the Gulf of Thailand belong to clade VII and VIII. There are 5 new haplotypes found in this study. Clade VII is also a unique clade for Thailand and Malaysia, especially CmP82. Gene flow between the green turtles in the Gulf of Thailand and the Malaysian and Brunei MUs and Vietnamese rookeries would be possible. The current results indicate that collaboration within South-east Asian countries (Thailand, Cambodia, Vietnam, Malaysia, Indonesia, and Brunei Darussalam) should be determined in future studies.

## Data Availability

Supplementary data can be available from the corresponding author on a reasonable request.

## Authors’ Contributions

PC and TS: Designed and conducted the study and wrote the manuscript. PC, WK, KS, NI, and TS: Participated in the scientific discussion and supervised the study. All authors have read, reviewed, and approved the final manuscript.
